# Lacunar stroke with and without chronic imaging biomarkers of small vessel disease: clinical profiles and stroke etiology

**DOI:** 10.3389/fneur.2026.1840105

**Published:** 2026-08-05

**Authors:** Jan Vynckier, Léonore Jaques, Basel Maamari, Martina Goeldlin, Lorenz Grunder, Michail Giannakakis, Elias Auer, Greta Sökeland, Janis Rauch, Bernhard Siepen, Philipp Bücke, Johannes Kaesmacher, Morin Beyeler, Arsany Hakim, Marcel Arnold, Bogdan Draganski, Jan Gralla, David J Seiffge, Urs Fischer, Thomas Raphael Meinel

**Affiliations:** 1 Department of Neurology, Inselspital Bern, Bern, Switzerland; 2 Department of Neurology, University Hospital Ghent, Ghent, Belgium; 3 Institute of Diagnostic and Interventional Neuroradiology, Inselspital, Bern University Hospital and University of Bern, Bern, Switzerland

**Keywords:** cerebral small vessel disease, lacunar stroke, magnetic resonance imaging, patent foramen ovale, vascular risk factors

## Abstract

**Introduction:**

Chronic cerebral small vessel disease (CSVD) may help distinguish different etiological mechanisms in acute lacunar stroke; this study aimed to compare vascular risk factors and stroke etiology in patients with and without CSVD.

**Methods:**

In this observational study, consecutive patients with magnetic resonance imaging (MRI)–confirmed acute lacunar stroke were included from a prospective stroke database covering the years 2015–2019. CSVD was defined as a summary small vessel disease (SVD) score ≥2 based on established MRI markers, including white matter hyperintensities, lacunes, cerebral microbleeds, and enlarged perivascular spaces. Associations between CSVD, vascular risk factors, and stroke etiology were examined using descriptive statistics and multivariable regression analysis.

**Results:**

Among 486 patients with acute lacunar stroke, 175 (36%) had CSVD. Median age was 72 years, 40.5% were female, and the median National Institutes of Health Stroke Scale (NIHSS) score on admission was 3. Patients with CSVD had a higher burden of vascular risk factors, including hypertension, previous stroke, diabetes, and atrial fibrillation, and presented with higher NIHSS scores and blood pressure on admission. A patent foramen ovale (PFO)–related stroke mechanism was less frequent in patients with CSVD (1.1% vs. 10.0%), although this association was not statistically significant in multivariable analysis (odds ratio 0.30, 95% CI 0.06–1.38).

**Discussion:**

A high SVD burden is consistent with hypertensive arteriopathy as the predominant underlying etiology, whereas minimal SVD may indicate alternative mechanisms such as PFO-related stroke. Recognition of these differences may support more tailored diagnostic evaluation and secondary prevention strategies.

## Introduction

Lacunar stroke accounts for roughly 20% of acute ischemic strokes and is one of the presenting syndromes of cerebral small-vessel disease (CSVD). Lacunar stroke is most often due to CSVD, however an acute small vessel occlusion can also occur due to other various etiologies, including embolism, hematological diseases and infectious or inflammatory arteritis (1–3).

Due to the limited diagnostic reliability of computed tomography (CT) imaging to accurately identify acute lacunar stroke and CSVD, (4) only few studies have investigated this subset of stroke patients. MR imaging is the gold standard in identifying both acute lacunar syndromes and CSVD, and enables accurate investigations (1). The hallmark features of CSVD on neuroimaging include lacunes, white matter hyperintensities (WMH), cerebral microbleeds and visible perivascular spaces (PVS) (5, 6). Based on these features, the severity can be estimated using the SVD sum score, which is a scale from 0 to 4, with higher total score indicating greater burden of disease (7).

It remains unclear which factors distinguish patients with acute lacunar stroke who exhibit radiological signs of significant CSVD from those without. We hypothesized that patients with acute lacunar stroke with moderate to severe CSVD would more frequently have vascular risk factors, as compared to lacunar stroke patients without chronic CSVD. Additionally, we hypothesized that those without chronic CSVD would more often have a non-SVD related stroke etiology. Thus, we aimed to compare vascular risk factors and etiology between MRI-proven lacunar stroke patients with and without chronic CSVD.

## Methods

### Patient population

The methodology has been described and published in detail before (8, 9). In short, this single-center study, based on MR imaging and conducted as a cross-sectional cohort investigation with a 90-day follow-up period, involved patients from the Swiss Stroke Registry who were consecutively admitted to the comprehensive university stroke center in Bern, Switzerland, between January 2015 and December 2019. To ensure diagnostic accuracy for acute lacunar stroke, only patients who underwent MRI upon admission were included. Patients with acute lacunar stroke were identified by reviewing MRI data manually. Lacunar stroke was defined as the presence of a recent small subcortical infarction within the territory of a perforating arteriole, (6) with no specific upper limit for the size (10). Patients with visible acute large or medium-sized vessel occlusions, multiple or cortical ischemic lesions, artery of Percheron infarction, (11) stroke mimics, and MRI-negative stroke were excluded.

### Clinical data collection

As part of the Swiss Stroke Registry, data were prospectively collected on pre-defined factors using electronic case report forms on a secured web-based database. From this database, we extracted various information, including demographics (age, gender, and pre-stroke modified Rankin Scale), cardiovascular risk factors (hypertension, smoking, dyslipidemia, diabetes), clinical parameters (i.e., systolic and diastolic blood pressure on admission, NIHSS scores at admission, prior use of medications like antithrombotic, antihypertensive and lipid-lowering drugs), as well as laboratory results (including glucose, cholesterol, and D-dimer levels). Stroke etiology is defined after a standardized diagnostic assessment including at least MR imaging of head and neck vessels, transthoracic echocardiography, 12-lead ECG, and 24 h of cardiac monitoring. A large proportion of patients also undergo transesophageal echocardiography as part of this standardized workflow. A modified Trial of Org 10,172 in Acute Stroke Treatment (TOAST) is used, which adds arterial dissection and PFO related stroke to the existing categories of the original TOAST criteria.

### Radiological assessment

All MRIs on admission were conducted using either a 1.5 or 3 Tesla MRI scanner from Siemens (MAGNETOM Avanto/Aera; MAGNETOM Vaerio/Skyra/Vida, Siemens, Erlangen, Germany). The standard stroke protocol encompassed, at a minimum, diffusion-weighted imaging, susceptibility-weighted imaging, and fluid-attenuated inversion recovery (FLAIR). All imaging data were reviewed and analyzed by a trained vascular neurologist (JV) and a neuroradiologist (LG).

The MRI biomarkers of small vessel disease were evaluated according to international consensus STRIVE criteria (6) and utilizing validated scales and scores: deep and periventricular white matter FLAIR hyperintensities (Fazekas scale), (12, 13) presence of cerebral microbleeds, (14) old lacunes, (15) and enlarged basal ganglia perivascular spaces. On this basis, the total small-vessel disease scores were calculated according to the score developed by Staals et al. (7) Raters were unaware of the study question of this manuscript at the timepoint of rating.

For the primary analysis, moderate to severe CSVD was defined as SVD sum score ≥2 with a sensitivity analysis using the cut-off of 1 for presence of any CSVD.

### Statistical analysis

Descriptive statistics were used to summarize baseline characteristics, stratified by the presence of moderate to severe CSVD. Continuous variables are presented as medians with interquartile ranges (IQR), and categorical variables as counts with percentages. Group comparisons were conducted using the table1_mc command in Stata, which applies Wilcoxon rank-sum tests for continuous variables and chi-squared tests for categorical variables. Univariate analyses compared demographic, clinical, and radiological characteristics between moderate to severe CSVD and non or minimal CSVD groups. Differences in imaging biomarkers were tabulated separately.

To assess associations between CSVD and presumed stroke etiology, we compared the distribution of modified TOAST classifications between groups using the chi-squared test. As a post-hoc analysis, logistic regression was used to evaluate the association between CSVD and presumed patent foramen ovale (PFO) etiology. The model adjusted for age, hypertension, prior TIA/stroke, diabetes and smoking history, as per components of the RoPE score (16, 17). Results are reported as adjusted odds ratios (aOR) with 95% confidence intervals (CI).

All statistical analyses were conducted using STATA (StataCorp. 2019. Stata Statistical Software: Release 16. College Station, TX: StataCorp LLC), including the table1_mc package. All *p*-values reported are two-sided, with *p* < 0.05 considered statistically significant, and no adjustments were made for multiple testing.

### Ethics

Ethical approval was obtained from the cantonal ethics committee (“Kantonale Ethikkommission” Bern) under the reference KEK 231/14, PB_2016–01905. The patient data utilized in this study were sourced from the Swiss Stroke Registry, which allows the enrollment of all patients treated at our stroke centers into a quality registry, in accordance with Swiss legislation (Law on highly specialized medicine—“HochspezialisierteMedizin” HSM; Art. 39 Abs. 2bis KVG). Patients who declined the usage of their data for scientific purposes were excluded from the analysis.

### Data availability

Anonymized data will be shared upon reasonably justified written request from any qualified investigator.

## Results

Of 4,611 patients admitted with an acute ischemic stroke during the study timeframe, 486 (10.5%) presented with a MRI-confirmed acute lacunar stroke. The median age was 72 years (IQR 61–80), 197 (40.5%) of patients were female and the median NIHSS score on admission was 3 (IQR 1–5).

In 175 (36.0%) of 486 patients presenting with acute lacunar stroke, there was evidence of moderate to severe CSVD (SVD sum score 2–4). These patients more often had severe white matter hyperintensities, with Fazekas grade 3 observed in 41.1% compared to only 1.6% in the non or minimal CSVD group. Lacunes and cerebral microbleeds were also more prevalent, present in 61.1 and 65.5%, respectively, versus 8.7 and 7.9% in the no CSVD group (see Table 1 for details on SVD biomarkers). A sensitivity analysis differentiating between complete absence of CSVD (SVD sum score 0) and any presence (SVD sum score ≥1) showed similar results (Supplemental Table S1).

**Table 1:** CSVD phenotypes of patients with lacunar stroke.

		Total *N* = 486	CSVD Sum score 0–1 (*N* = 311)	CSVD Sum score 2–4 (*N* = 175)
White matter hyperintensities (Fazekas)	0	151 (31.2%)	143 (46.3%)	8 (4.6%)
	1	134 (27.7%)	103 (33.3%)	31 (17.7%)
	2	122 (25.2%)	58 (18.8%)	64 (36.6%)
	3	77 (15.9%)	5 (1.6%)	72 (41.1%)
Lacunes	At least one	134 (27.6%)	27 (8.7%)	107 (61.1%)
Cerebral microbleeds	At least one	138 (28.8%)	24 (7.9%)	114 (65.5%)
Enlarged perivascular spaces in the basal ganglia	0	197 (40.8%)	174 (56.5%)	23 (13.1%)
	1	189 (39.1%)	88 (28.6%)	101 (57.7%)
	2	43 (8.9%)	31 (10.1%)	12 (6.9%)
	3	41 (8.5%)	12 (3.9%)	29 (16.6%)
	4 or more	13 (2.7%)	3 (1.0%)	10 (5.7%)
CSVD sum score	0	192 (39.5%)	192 (61.7%)	0 (0.0%)
	1	119 (24.5%)	119 (38.3%)	0 (0.0%)
	2	88 (18.1%)	0 (0.0%)	88 (50.3%)
	3	57 (11.7%)	0 (0.0%)	57 (32.6%)
	4	30 (6.2%)	0 (0.0%)	30 (17.1%)

Data are presented as *n* (%). CSVD, cerebral small vessel disease, N, number (of participants).

In univariate analysis, patients with moderate to severe CSVD more often had a history of hypertension (90.3% vs. 70.1%), a history of previous stroke (24.6% vs. 10.3%), diabetes (25.1% vs. 16.4%), and atrial fibrillation (18.9% vs. 11.3%)—see Table 2 for full baseline characteristics. A substantially higher proportion was pre-treated with lipid-lowering medication (30.9% vs. 19.3%), whereas use of antiplatelet or anticoagulant medication did not differ between the two groups. While blood sample values such as cholesterol, HbA1C, glucose and creatinine did not differ, clinical presentation was worse in the moderate to severe CSVD group with higher median NIHSS (4 (2–6) vs. 3 (1–5)) and higher systolic and diastolic blood pressure values on admission (179 (162–204) mmHg vs. 167 (145–189) mmHg; 90 (80–101) mmHg vs. 87 (75–99) mmHg).

**Table 2:** Baseline variables.

	Total (*N* = 486)	CSVD Sum score 0–1 (*N* = 311)	CSVD Sum score 2–4 (*N* = 175)	*p* value
Demographics				
Age (years)	72 (61–80)	68 (56–77)	76 (68–84)	<0.001
Female sex	197 (40.5%)	127 (40.8%)	70 (40%)	0.86
Pre-stroke modified Rankin scale	0 (0–0)	0 (0–0)	0 (0–1)	<0.001
Medical history				
Previous stroke	75 (15.4%)	32 (10.3%)	43 (24.6%)	<0.001
Previous transient ischemic attack	36 (7.4%)	23 (7.4%)	13 (7.4%)	0.99
Previous intracerebral hemorrhage	4 (0.8%)	2 (0.6%)	2 (1.1%)	0.56
Hypertension	376 (77.4%)	218 (70.1%)	158 (90.3%)	<0.001
Smoker	126 (26%)	84 (27%)	42 (24%)	0.47
Diabetes	95 (19.5%)	51 (16.4%)	44 (25.1%)	0.020
Dyslipidemia	337 (69.3%)	219 (70.4%)	118 (67.4%)	0.49
Coronary heart disease	60 (12.3%)	33 (10.6%)	27 (15.4%)	0.12
Peripheral arterial disease	18 (3.7%)	8 (2.6%)	10 (5.7%)	0.078
Atrial fibrillation	68 (14%)	35 (11.3%)	33 (18.9%)	0.020
Prior medication				
Antiplatelet	153 (31.4%)	90 (29%)	63 (36%)	0.25
Anticoagulant	52 (10.7%)	27 (8.7%)	25 (14.3%)	0.35
Lipid-lowering drugs	114 (23.5%)	60 (19.3%)	54 (30.9%)	0.004
Clinical features				
National Institute of Health Stroke Scale score on admission	3 (1–5)	3 (1–5)	4 (2–6)	0.027
Capsular warning syndrome	41 (8.4%)	24 (7.7%)	17 (9.7%)	0.447
Type of lacunar syndrome				0.685
- Pure motor stroke	76 (15.6%)	50 (16.1%)	26 (14.9%)	
- Ataxic hemiparesis	8 (11.9%)	36 (11.6%)	22 (12.6%)	
- Pure sensory stroke	20 (4.1%)	15 (4.8%)	5 (2.9%)	
- Sensoriataxic	17 (3.5%)	13 (4.2%)	4 (2.3%)	
- Sensorimotor	68 (14.0%)	45 (14.5%)	23 (13.1%)	
- Dysarthria clumsy hand	5 (1.0%)	4 (1.3%)	1 (0.6%)	
Systolic blood pressure on arrival (mmHg)	170 (151–191)	167 (145–189)	179 (162–204)	<0.001
Acute treatment				
Intravenous thrombolysis	114 (23.5%)	87 (28%)	54 (30.9%)	0.50

Data are presented as median (IQR) for continuous measures and *n* (%) for categorical measures. CSVD, cerebral small vessel disease; N, Number (of participants).

The distribution of the presumed stroke etiology according to the modified TOAST classification differed significantly between patients with and without moderate to severe CSVD (*p* = 0.007; Table 3). SVD was the most common etiology in both groups (63.7% in patients without CSVD and 65.1% with CSVD). Cardiac embolism was more frequent in the CSVD group (13.1% vs. 8.4%), while patent foramen ovale (PFO) related stroke was notably less common in patients with CSVD (1.1% vs. 10.0%). Other etiologies, including large artery atherosclerosis, multiple possible causes, and other determined rare causes, were similarly distributed across groups.

**Table 3:** Presumed most likely modified trial of org 10,172 in Acute Stroke Treatment (TOAST) etiologies in patients with lacunar stroke according to imaging presence of chronic CSVD.

Etiology	Total (*N* = 486)	No CSVD (*N* = 311)	CSVD (*N* = 175)	*P* value
				0.007
Cardiac embolism	49 (10.1%)	26 (8.4%)	23 (13.1%)	
Large artery atherosclerosis	23 (4.7%)	14 (4.5%)	9 (5.1%)	
More than one possible etiology	57 (11.7%)	34 (10.9%)	23 (13.1%)	
Other determined etiology	12 (2.5%)	8 (2.6%)	4 (2.3%)	
PFO	33 (6.8%)	31 (10.0%)	2 (1.1%)	
Small vessel disease	312 (64.2%)	198 (63.7%)	114 (65.1%)	
Arterial dissection	0 (0%)	0 (0%)	0 (0%)	

CSVD, cerebral small vessel disease defined as SVD sum score of 2–4 points. Although arterial dissection was part of the categories, none of the patients was in this category, thus it was omitted from the model.

When adjusting for the components of the RoPE score, CSVD was not independently associated with presumed PFO-related etiology (aOR 0.30, 95% CI 0.06–1.38) in an explanatory post-hoc analysis.

## Discussion

In this MRI-based study in patients with acute lacunar stroke, approximately one-third exhibited a moderate-to-severe CSVD burden. These patients showed a classical CSVD risk profile, with a notably high prevalence of hypertension, prior stroke, and diabetes. This finding aligns with earlier work showing a strong association between vascular risk factors—especially systolic hypertension—and chronic CSVD (18). Interestingly, atherosclerotic risk factors such as smoking and dyslipidemia were similarly distributed across groups, reinforcing the importance of hypertensive arteriopathy in CSVD pathogenesis.

A post-hoc finding of this study was a potential difference in presumed stroke etiology between patients with and without moderate to severe CSVD. While SVD was evidently the most common classification in both groups, presumed PFO-related strokes were several times more frequent among those without CSVD. The effect was somewhat attenuated after adjustment for variables included in the RoPE score, but the point estimate did indicate a potentially meaningful association—which is also pathophysiologically plausible. Our study was underpowered for this question, so this should be considered hypothesis-generating. The findings align with the etiologic heterogeneity of lacunar stroke: most cases reflect intrinsic small vessel pathology, but a relevant minority might be caused by embolic sources, including paradoxical embolism through a PFO (1, 2). Our results—if confirmed—suggest that in patients with acute lacunar infarcts but no or minimal CSVD on MRI—particularly younger patients with a low vascular risk factor burden—a thorough diagnostic work-up with adequate assessment for PFO could be considered (2, 19).

Interestingly, cardioembolic strokes due to atrial fibrillation were slightly more frequent in the moderate to severe CSVD group. This probably reflects shared risk factors for atrial fibrillation such as hypertension and age rather than stroke mechanism per se and highlights that multiple pathologies may coexist (20). Thus, stroke etiology should not be determined solely by infarct pattern or imaging appearance, but by integrating risk profiles and targeted investigations. These can be complemented by CSVD burden assessment using MRI. Prior work by Staals et al. and others introduced the SVD sum score as a practical tool to quantify CSVD (7). Our study supports that approach, in capturing clinically meaningful CSVD burden, allowing for individualization of stroke care for lacunar stroke patients. High SVD burden is not only a marker of intrinsic small vessel pathology but also associated with early neurological deterioration, (21) poorer early recovery, (22) cognitive impairment, (23) depression (24) and increased risk of stroke recurrence (25).

In line with other studies, which demonstrated the progressively increasing use of MRI in lacunar stroke research and emphasized the heterogeneity of lacunar infarction etiologies, our MRI-based study extends these observations by showing that patients with acute lacunar infarction without chronic imaging biomarkers of CSVD exhibit a distinct vascular risk factor and etiologic profile compared with those with substantial CSVD burden, supporting the concept that MRI characterization of chronic small vessel injury may help identify biologically different lacunar stroke subgroups (7, 8, 25, 26). Patients with significant CSVD might benefit from intensified vascular risk factor control and closer follow-up for cognitive and functional decline. In contrast, acute lacunar stroke patients with no or minimal CSVD warrant careful etiologic evaluation—including dedicated cardiac investigations screening both for atrial fibrillation and for a PFO—since their infarct may not stem from arteriolosclerosis and specific treatments are available to reduce the risk of stroke in these patients.

Similar to acute lacunar ischemic stroke, future studies should investigate whether the presence or absence of chronic imaging biomarkers of CSVD also identifies biologically and clinically distinct subgroups among patients with hemorrhagic stroke, particularly deep intracerebral hemorrhage, as prior work suggests that ischemic and hemorrhagic manifestations of cerebral small vessel disease differ in vascular risk factors, clinical presentation, and outcomes despite sharing a common arteriolar substrate (27).

### Strengths and limitations

A key strength of our study is the large and well-characterized patient cohort included in our database. The use of multimodal MRI provides strong diagnostic confidence that the observed strokes were indeed due to isolated (single) small perforator vessel infarctions. Despite the retrospective study design, the prospective data acquisition and highly standardized workflow for both the management and etiological workup contribute to the reliability and robustness of our findings. Moreover, CSVD burden using SVD sum score was assessed by experienced vascular neurologists using internationally established rating criteria, ensuring reliable and consistent scoring.

However, there are several limitations to mention. First, due to its retrospective design, there is a possibility of selection bias—including patients not able to undergo MRI during hospitalization. Compared to population-based cohorts, the proportion of lacunar stroke patients at our center is relatively low (10%). This is mainly because it is a comprehensive stroke center, where many large vessel occlusion patients are referred to for acute invasive interventions such as mechanical thrombectomy. The inclusion of all consecutive patients somewhat mitigates the risks of selection bias. Second, even though a significant association was found between hypertension and small vessel disease, a large proportion of patients without CSVD also had pre-existing hypertension. Due to its retrospective nature, history taking or medical file recordings of hypertension are not always complete and might even be less looked for in patients without evidence of CSVD on imaging. Third, as the modified TOAST criteria for stroke etiology depend on individual estimation of causality, as well as performed technical investigations, we cannot exclude a self-fulfilling effect of the presence of a higher total SVD burden score in patients and the physician’s ultimately concluding that the acute stroke was indeed caused by SVD. This may have led to a relative underestimation of competing etiologies in exactly those patients. However, in our institution and in this cohort, more than 85% of patients received a full diagnostic work-up and moreover, patients in whom competing etiologies are found to be present, are identified as unknown etiology because of these multiple possible etiologies for the index event. Lastly, the comparisons were not adjusted for multiple testing since our intention was to characterize clinical and etiologic differences between imaging-defined subgroups of lacunar stroke patients—thus the study is primarily exploratory and hypothesis-generating^1^.

## Conclusion

In summary, CSVD markers help distinguish clinically meaningful subgroups among patients with acute lacunar stroke. Those with significant CSVD burden likely represent the classical phenotype of hypertensive small vessel disease. In contrast, patients with minimal or no CSVD exhibit a different risk profile and may have an alternative stroke mechanism such as PFO-related stroke. Future prospective multicenter studies integrating standardized etiologic workup, advanced imaging biomarkers, and longitudinal outcome assessment are needed to determine whether CSVD-guided stratification can improve individualized secondary prevention strategies in lacunar stroke.

## Data Availability

The original contributions presented in the study are included in the article/Supplementary material, further inquiries can be directed to the corresponding author.

## Footnotes

1 Jung S Mattle H Horvath T et al. Stroke Guidelines of the Bern Stroke Network 2021 http://www.neurologie.insel.ch/fileadmin/Neurologie/Dokumente/Stroke_Center/Stroke_Guidelines_2021_English.pdf
