# Supplementary material for: Lacunar stroke with and without chronic imaging biomarkers of small vessel disease: clinical profiles and stroke etiology
Source: Front Neurol. 2026 Aug 5;17:1840105. doi: 10.3389/fneur.2026.1840105 (PMC13487495; doi:10.3389/fneur.2026.1840105)
Supplement: Supplementary file 1 [file Table_1.docx]

**Supplemental Material**

|  | Total  (N = 486) | CSVD Sum score 0  (N = 192) | CSVD Sum score 1-4  (N = 294) | P value |
| --- | --- | --- | --- | --- |
| Demographics |  |  |  |  |
| Age (years) | 71.5 (60.6-80.1) | 65.9 (54.6-75.5) | 74.3 (65.4-81.2) | <0.001 |
| Female sex | 197 (40.5%) | 75 (39.1%) | 122 (41.5%) | 0.59 |
| Pre-stroke modified Rankin scale | 0 (0-0) | 0 (0-0) | 0 (0-1) | <0.001 |
|  |  |  |  |  |
| Medical history |  |  |  |  |
| Previous stroke | 75 (15.4%) | 17 (8.9%) | 58 (19.7%) | 0.001 |
| Previous transient ischemic attack | 36 (7.4%) | 10 (5.2%) | 26 (8.8%) | 0.13 |
| Previous intracerebral hemorrhage | 4 (0.8%) | 1 (0.5%) | 3 (1.0%) | 0.55 |
| Hypertension | 376 (77.4%) | 127 (66.1%) | 249 (84.7%) | <0.001 |
| Smoker | 126 (25.9%) | 56 (29.2%) | 70 (23.8%) | 0.19 |
| Diabetes | 95 (19.5%) | 33 (17.2%) | 62 (21.1%) | 0.29 |
| Dyslipidemia | 337 (69.3%) | 134 (69.8%) | 203 (69.0%) | 0.86 |
| Coronary heart disease | 60 (12.3%) | 17 (8.9%) | 43 (14.6%) | 0.059 |
| Peripheral arterial disease | 18 (3.7%) | 4 (2.1%) | 14 (4.8%) | 0.13 |
| Atrial fibrillation | 68 (14.0%) | 18 (9.4%) | 50 (17.0%) | 0.018 |
|  |  |  |  |  |
| Prior Medication |  |  |  |  |
| Antiplatelet | 147 (30.2%) | 52 (27.1%) | 95 (32.3%) | 0.43 |
| Anticoagulant | 52 (10.7%) | 14 (7.3%) | 38 (12.9%) |  |
| Lipid-lowering drugs | 114 (23.5%) | 38 (19.8%) | 76 (25.9%) | 0.12 |
|  |  |  |  |  |
| Clinical features |  |  |  |  |
| National Institute of Health Stroke Scale score on admission | 3 (1-5) | 3 (1-5) | 3 (1-5) | 0.009 |
| Systolic blood pressure on arrival (mmHg) | 170 (151-194) | 162 (143-184) | 175 (157-199) | <0.001 |
|  |  |  |  |  |
| Acute treatment |  |  |  |  |
| Intravenous thrombolysis | 141 (29.0%) | 44 (22.9%) | 97 (33.0%) | 0.017 |

**STable 1 Baseline comparison for the sensitivity analysis**. Data are presented as median (IQR) for continuous measures and n (%) for categorical measures.
CSVD – Cerebral Small Vessel Disease, N – Number (of participants)
